# Genome-Wide Comparative Analysis of Miniature Inverted Repeat Transposable Elements in 19 *Arabidopsis thaliana* Ecotype Accessions

**DOI:** 10.1038/s41598-017-02855-1

**Published:** 2017-06-01

**Authors:** Cheng Guo, Matthew Spinelli, Congting Ye, Qingshun Q. Li, Chun Liang

**Affiliations:** 1 Department of Biology, Miami University, Oxford, OH 45056 USA; 2 Key Laboratory of the Ministry of Education for Costal and Wetland Ecosystems College of the Environment and Ecology, Xiamen University, Xiamen, Fujian 361102 China; 3 Graduate College of Biomedical Sciences, Western University of Health Sciences, Pomona, CA 91766 USA

## Abstract

Miniature inverted repeat transposable elements (MITEs) are prevalent in eukaryotic genomes. They are known to critically influence the process of genome evolution and play a role in gene regulation. As the first study concentrated in the transposition activities of MITEs among different ecotype accessions within a species, we conducted a genome-wide comparative analysis by characterizing and comparing MITEs in 19 *Arabidopsis thaliana* accessions. A total of 343485 MITE putative sequences, including canonical, diverse and partial ones, were delineated from all 19 accessions. Within the entire population of MITEs sequences, 80.7% of them were previously unclassified MITEs, demonstrating a different genomic distribution and functionality compared to the classified MITEs. The interactions between MITEs and homologous genes across 19 accessions provided a fine source for analyzing MITE transposition activities and their impacts on genome evolution. Moreover, a significant proportion of MITEs were found located in the last exon of genes besides the ordinary intron locality, thus potentially modifying the end of genes. Finally, analysis of the impact of MITEs on gene expression suggests that migrations of MITEs have no detectable effect on the expression level for host genes across accessions.

## Introduction

Miniature inverted repeat transposable elements (MITEs) are a group of non-autonomous DNA transposons, which are prevalent in most eukaryotic genomes^1–5^. A complete MITE is a short DNA sequence that is characterized by a pair of **t**erminal **i**nverted **r**epeats (TIRs, ≥10 *nt*), and flanked by a pair of direct repeat sequences known as **t**arget **s**ite **d**uplications (TSDs, 2~10 *nt*). MITEs are normally short sequences with lengths ranging from 50 to 800 *nt*, and in some studies MITEs are even referred as a relatively short (<800 *nt*) DNA transposons regardless of its sequence feature or copy number in the genome^6^. Unlike other DNA transposons with only a limited number of copies in the genome, hundreds of MITE families and family units were found and classified across species^2, 7, 8^. Considering their tremendous copy numbers, MITE transpositions could result in considerable changes affecting genome structure and phenotypic diversity and plasticity. Similar MITEs are first grouped into families based on sequence similarity, and then into superfamilies primarily characterized by identical or similar TIR and TSD sequences^9–12^. The first classified MITE family is the *Tourist*, which was found in maize a few decades ago^1^. *Tourist* MITEs presented themselves as a novel transposable element (TE) class not only because they represented a group of sequences bearing conserved 14 *nt* TIRs and flanked by 3 *nt* TSDs, but also no sequence similarity was found between them and any other known TEs at that time. After revealing the *Tourist*, a large variety of MITEs were detected and classified^2^. Currently, a widely accepted MITE classification in plant genomes contains the following seven superfamilies: *Tcl/mariner*, *PIF/Harbinger*, *hAT*, *Mutator*, *CACTA*, *P-element* and *Novosib* superfamily, while a total of 15 different superfamilies are reported for all known DNA transposon elements^13–15^. For instance, *Tourist* MITEs were classified as a family in the *PIF/Harbinger* superfamily. Focusing on MITEs in plants, the P-MITE database collected putative MITE sequences to elucidate their origins and amplification activities during evolution in 41 plant species. The resulting database contains 2.3 million MITE sequences in total, which were further classified into 3527 families and 7 superfamilies. A positive correlation was found between MITE abundance with genome size, indicating an association between MITEs and genome expansion^13^. Along with other similar studies^16–19^, the P-MITE database provided a comprehensive and valuable resource to TEs-related studies in plants.

MITEs have been considered as defective derivatives of other autonomous transposable elements^20, 21^. Since internal deletion/mutation left their transposase encoding capacity diminished during evolution, their amplification is largely dependent on the availability and activity of trans-acting transposases produced by their corresponding autonomous TEs^22, 23^. These specific transposases catalyze amplification and transposition of MITEs by recognizing common TIR sequences^24–26^. Nonetheless, most MITE families are inactive while the occasionally identified transposition activities of MITEs are usually associated with external stresses^27^. Intriguingly, MITEs feature a much higher copy number than other DNA transposons in genomes, despite the fact that they are all sharing the same transposases catalyzing their amplification and transposition. It is still obscure why MITEs are more successful in amplification than their progenitors, considering their own internal resource of transposase has diminished during evolution. Hypotheses attempting an explanation include the secondary structure formed in MITEs leads to a better affinity with the transposase, or the deletion of a sequence undermines the blocking influence via DNA methylation^23, 28^. However, the mechanism underlying the acquisition, loss, and high copy number of MITEs are still vague and the systematical study of these open questions is lacking.

In plants, MITEs often reside in gene-rich regions, such as introns or intergenic regions close to gene ends. For example, the majority of MITEs from the *Monkey King* family are located within 3 kbps of a gene in *A. thaliana* and the relative frequency of MITEs is greater as the they get closer to genes^16^. MITEs were shown to fully or partially contribute to the transcription starting site, splicing junction and poly(A) site for some genes^9, 29^. Meanwhile, the preferred location and potential impact of MITEs vary in different families as well. MITEs are generally recruited inside introns^30^, while it also has been reported that *BraTo-9*, a *Tourist* family of MITEs from *Brassica rapa*, is preferentially present in the exons of triplicated *Brassica rapa* genes^18^. On the other hand, studies suggesting the role of MITEs in the gene expression regulation were reported in a variety of organisms^31–33^. In rice, the impact of MITEs on gene expression is significant, with more than 7,000 MITEs transcribed in transcription, and about half of them are transcribed along with protein coding sequences^9^. A genome-wide study suggested that genes with embedded or nearby MITEs are likely to present lower expressions compared to the genes without MITE-gene interaction^34^. This down-regulation may be caused by MITE-derived small RNAs since the inverted TIR sequences could form double-stranded RNAs when transcribed, hence becoming the resource for miRNA generation^35^. Indeed, more than 23.5% of small RNAs in rice^34^.

The function of a MITE in individual gene regulation has been occasionally reported. For instance, the MITE within the promoter region of the ubiqutin2 gene (*RUBQ2*) increased the gene expression level in rice, and such enhanced expression is neutralized by DNA methylation of the MITE sequence^36^. Another study suggested that a MITE, located upstream of the *SbMATE* gene, operated as a *cis*-acting element to up-regulate *SbMATE*, introducing a positive correlation between the presence of the repeat structure and aluminum tolerance^37^. A MITE insertion in the 3′ UTR of the *TaHSP16.9–3A* gene in wheat was shown to greatly increase the transcription level under heat stress compared to the other wheat haplotypes without the MITE insertion, hence resulting in heat tolerance^38^. As with other types of transposable elements, MITE sequences are also normally associated with heavy DNA methylation, which provides another layer for gene regulation^39, 40^.

With the rapidly growing number of sequenced genomes and the development of computational tools for MITE detections, the number of identified MITEs have dramatically increased^13, 41–44^. Despite this, many aforementioned questions remain open, including the role that MITEs play in gene expression regulation and genome evolution. Many efforts have been devoted to answer these questions, however, either analyses were limited to individual species, or the genomic comparative analyses were constrained within interspecies level to interpret the impact of MITE on chromosome reorganization^16, 18, 45, 46^. In this study, a novel genome-wide intra-species comparative analysis was conducted by characterizing and comparing MITEs in 19 *Arabidopsis* accessions. We delineated a total of 343485 putative MITE sequences from the 19 accessions. Among them, 4207 are canonical MITE sequences (with perfect and near perfect TSD/TIR sequences) and other non-canonical ones are either diverse or partial ones. Within the entire population of those MITEs, 80.7% of them were previously unclassified MITEs and showed to have a different genomic distribution and functionality compared to the classified MITEs in P-MITE database. The interactions between MITEs and homologous genes across 19 accessions provided a fine source for analyzing MITE transposition activity and the relevant impact on gene expression and genome evolution in an intra-species perspective. Additionally, besides the canonical intron locality^47^, a significant proportion of MITEs were found to be located in the last exon of genes, thus potentially modifying the ends of genes. Lastly, analysis of the impact of MITEs on gene expression suggested that overall expression of genes associated with MITEs is significantly higher than that of genes without MITE interaction; however, no detectable effect was found on an individual gene level.

## Methods

### Data and tools used in the study

The whole genome sequences of 19 accessions of *A. thaliana* and their gene annotation information were obtained from http://mtweb.cs.ucl.ac.uk/mus/www/19genomes/ ^48^. These 19 accessions represented the parents of more than 700 MAGIC (Multiparent Advanced Generation Inter-Cross) lines^49^ and covered a fairly good geographical and phenotypical diversity across the *A. thaliana* population. Details about the 19 accessions (named as *col*, *bur*, *can*, *ct*, *edi*, *hi*, *kn*, *ler*, *mt*, *no*, *oy*, *po*, *rsch*, *sf*, *tsu*, *wil*, *ws*, *wu* and *zu* in our study) are listed in Supplementary Table 1. Besides the *col* (TAIR10) accession, the other 18 have assembled genomes provided by the *Arabidopsis* 1001 project (http://1001genomes.org/). Their genomic annotations are bond-fide since less than one assembly error was found per gene^49^. The consolidated gene annotation across accessions was made in the original study through integrating together the gene models predicted by the whole transcriptomic data and by *ab initio* prediction methods^48^. As a consequence of *ab initio* prediction, the homologous genes were named consistently following the conventions in TAIR10^50^. If any of the predicted genes were not recorded in TAIR10, they were classified as novel genes. Meanwhile, the processed transcriptomic data from the same study was also downloaded to examine the impact that MITEs have on gene expression. The plant material used for RNA-seq library preparation is fresh seedling tissues, which is the same material as used for genome sequencing. Representative MITE sequences for classified MITE families and superfamilies in 41 plant genomes were obtained from the P-MITE database and used as the references to represent MITE family and superfamily information in our study. To note, the representative sequences by *de novo* detection in our result were grouped as unclassified MITEs, if not found associated with any record in the P-MITE database using sequence similarity search.

### Identification of MITEs

The canonical MITE sequences of the 19 accessions were separately detected by detectMITE, a tool developed by us recently^43^. The detectMITE program requires the canonical MITE sequences to meet the structural constraint (perfect TSDs and mostly perfect TIRs) and the copy number constraint (*i.e*., at least 3 full-length copies in the genome with distinctive flanking sequences)^43^. Though the position and size information of TSD sequences were available in detectMITE output, the actual TSD sequences were not included within the output. Hence the *get-genome* program in GMAP^51^ was used to extract the flanking TSD sequences to MITEs based on the TSD location information provided by detectMITE. In order to classify all MITE sequences (individual canonical MITE sequences reported by detectMITE) into families and superfamilies, all complete canonical MITE sequences from the 19 accessions were pooled into a single FASTA file and clustered using CD-HIT^52^. The canonical MITE sequences having at least 80% sequence similarity were clustered. The characterizations of canonical MITE sequences, including GC content and TSD/TIR sequence length, were parsed by custom-built PERL scripts. To annotate these canonical MITEs with MITE family and superfamily information, the representative sequences from all CD-HIT cluster groups (*i.e*., the longest MITE sequence in each cluster group) were aligned by RepeatMasker against a custom-defined library using the default settings (-s -nolow -norna -no_is)^53^, which contained all known/classified MITE sequences from 41 plant species downloaded from the P-MITE database with proper MITE family and superfamily annotations^13^. The family and superfamily information was inherited if a sequence was aligned with any record in the P-MITE database, while the rest, orphaned sequences which were not aligned, were grouped into the category of unclassified MITEs. Complete canonical (perfect or near perfect) MITE sequences only represent a small portion of the MITE population, thus RepeatMasker with the default setting was applied again to identify all putative MITEs in all 19 accessions, including diverse and partial ones as well, utilizing the annotated list of complete canonical representative MITEs as the custom-defined library. The final MITEs database is available through link (https://github.com/gc26762524/MITEsin19Arabidopsis).

### MITE insertion polymorphism analysis

As the homologous genes were consistently annotated among all 19 accessions, these MITE-related homologous genes provide a great resource of genetic markers for analysis of MITE insertion polymorphism (MIP). To find out the association between genes and MITEs, the distances between MITEs and their closest gene were calculated based on their relative locations in genomes. The criteria defining whether a MITE was associated with a gene required their distance to be less than 40 *nt*. The MITEs were further annotated to check if they were located in the 5′ UTR, coding exonic region, coding intronic region, 3′ UTR or in the intergenic regions. Meanwhile, MITEs were specifically annotated if they spanned over more than one genetic sub-regions (*i.e*., 5′ UTRs, 3′ UTRs, CDS introns or CDS exons). By comparing the relative locations of MITEs in homologous genes among all 19 accessions, the transposition activities of MITEs were examined by customized R and PERL scripts. A MySQL database was built using Python to store the position and characterization information of both MITEs and their associated genes. Additional information was also included in the database, such as basic characterization of genes and MITEs and so on. Snapshots of the relative locations of associated MITEs and genes were obtained from GBrowser^54^ and IGV^55^.

### Impact of MITEs on gene expression

Using the processed transcriptomic data of the 19 accessions obtained from the same original study^48^, RPKM values for gene expression were extracted and only the genes having association with MITEs in at least one accession were considered. A gene might be correlated with MITEs in some accessions while not in others because of differential MITE transposition during evolution, hence the comparison of relative expression levels between a gene with and without a MITE insertion among different accessions could help identify the direct impact of a MITE on a gene’s expression level. After normalizing all 19 accessions, RPKM values for genes between the two groups (with and without MITE) were collected and compared with Mann-Whitney U test (P-value < 0.05) in R^56^.

## Results

### Characterization of MITE families in 19 accessions

**Figure 1:**
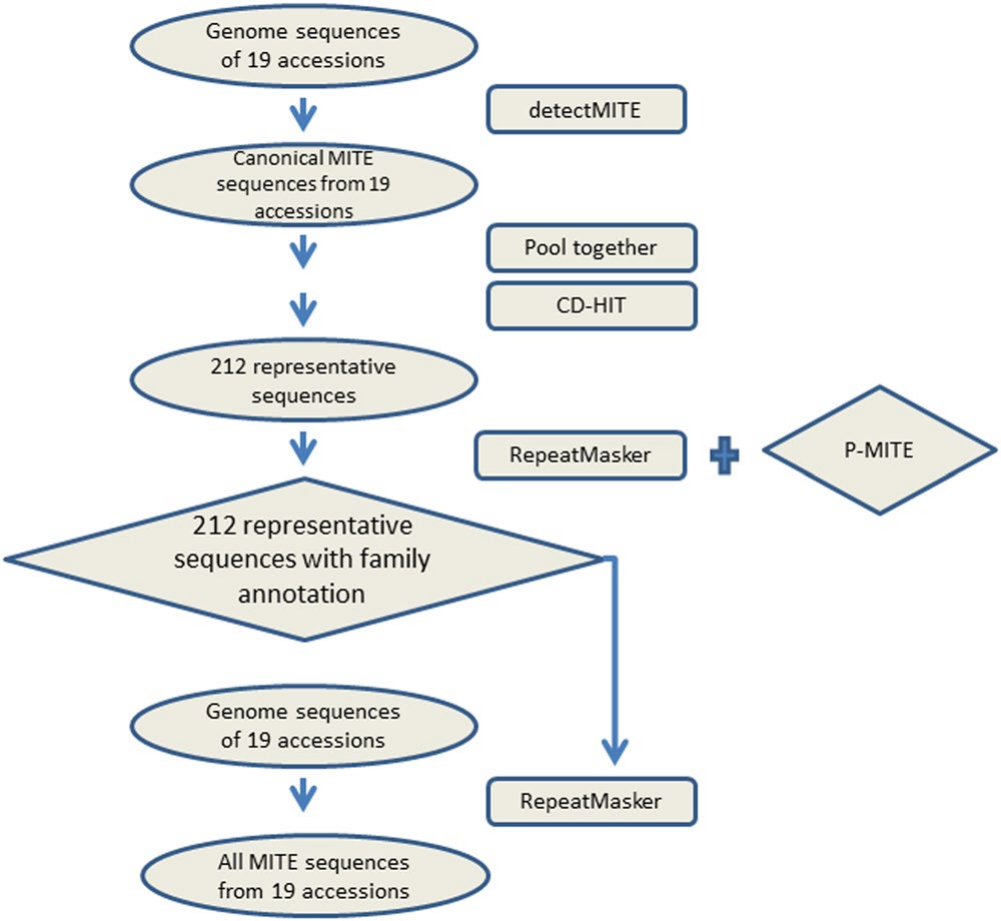
Flowchart of the pipeline for MITE identification in 19 *Arabidopsis thaliana* accessions.

The current approaches of identifying MITEs in genomes consist of two major steps: (1) the *de novo* identification of canonical MITE sequences (or using a pre-existing database of MITEs without *de novo* detection), and (2) the homologous search using the canonical MITE sequences as the seeds to uncover more MITE sequences^13^. Slightly different from such conventional approaches, the flowchart of our pipeline is illustrated in Fig. 1. To begin with, the genome-wide *de novo* detections of putative canonical MITEs in all 19 ecological accessions of *A. thaliana* were conducted by detectMITE^43^. Canonical MITE sequences with perfect TSD and perfect or near perfect TIR structure, whose length were between 50 to 800 *nt*, were identified in all 19 accessions separately. Averaging 221.4 canonical MITEs per accession, the most and least MITE-contained accessions were *col* (293) and *edi* (195). The lengths of these canonical MITEs ranged from 54 to 794 *nt* with an average of 289 *nt*. The lengths of TIRs ranged from 10 to 56 *nt* with an average of 14.1 *nt*, meanwhile the length of TSDs ranged from 2 to 10 *nt* with an average of 2.9 *nt*. A more detailed description of these canonical MITE sequences in 19 accessions is recorded in Supplementary Table 1.

To our best knowledge, previous MITE studies were limited within interspecies comparison, and the detection of MITEs of a species was solely based on its own genome refs 13, 41. However, such approach may result in a big underestimation of the abundance of canonical MITEs used for homology search compared to our approach, which pooled all canonical MITE sequences from 19 accessions for the homology search. MITE sequences are not necessary to have perfect TSD and TIR sequences for transposition, and individual site mutations (e.g. indels) or polymorphism within the TSD/TIR region may easily ruin the canonical structure. To minimize such impact, the canonical MITE sequences from all 19 accessions were pooled together, and then clustered based on the sequence similarity, regardless of which accession the MITEs were from. A total of 212 MITE groups were obtained from the pooled 2406 canonical MITE sequences across 19 accessions using CD-HIT, as suggested by DetectMITE. The longest sequence in each group was designated as the representative sequence. Representative sequences were further annotated with the superfamily/family information by aligning to the P-MITE database using RepeatMasker with default settings. Among these 212 representative sequences, 91 were annotated with known superfamily information, namely *hAT* (6 families were detected in the *hAT* superfamily), *PIF/Harbinger* (15 families were detected in the *PIF/Harbinger superfamily*), *Mutator* (10 families were detected in the *Mutator* superfamily) and *Tc1/Mariner* (7 families were detected in the *Tc1/Mariner* superfamily). In contrast, the remaining 130 representative sequences were found not to be related with any P-MITE annotation, and thus named as unclassified MITE clusters. To note, the *A. thaliana* MITE sequences in the P-MITE database only contained these four superfamilies as well. In addition, all 19 accessions presented similar proportion of superfamilies/families, suggesting relatively stable MITE compositions, as no specific superfamilies/families atypically expanded during the evolution process (Supplementary Table 1).

**Figure 2:**
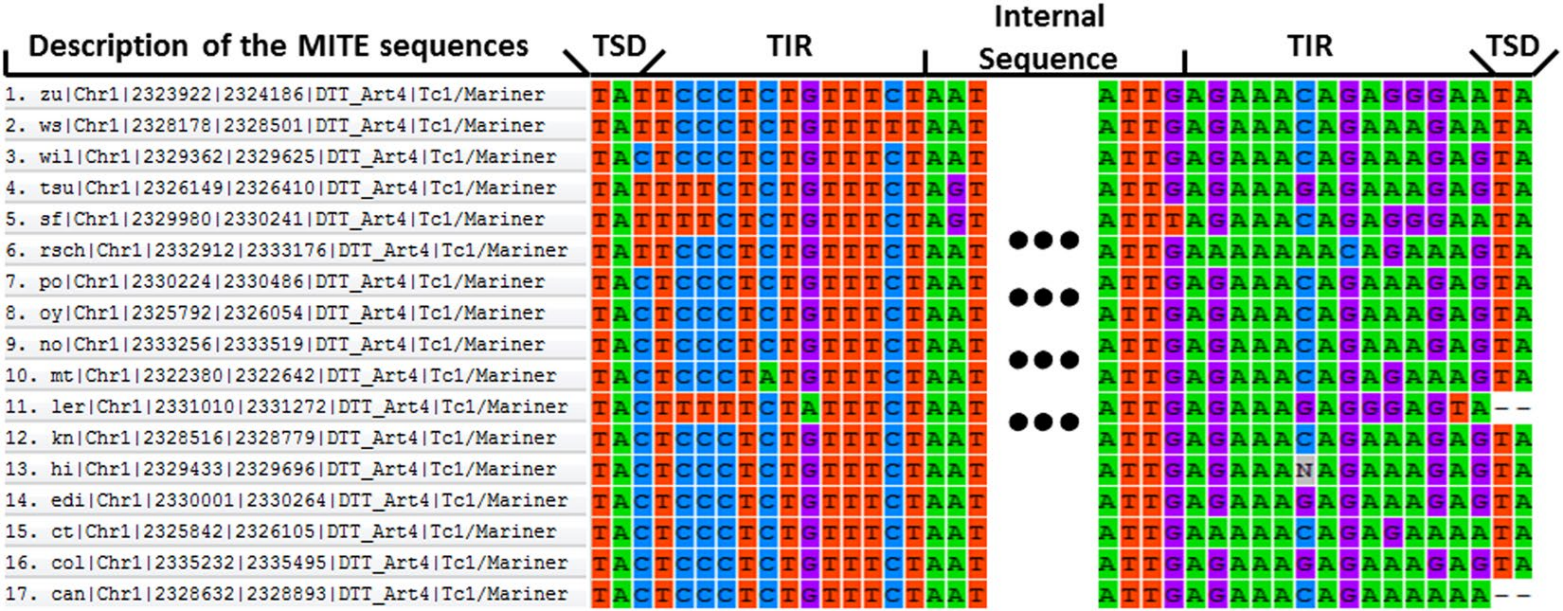
Multiple sequence alignment for a homologous MITE group by MEGA6. The description of the MITE sequences column includes the basic MITE information, placed in descending order of accession name, chromosome name, starting position, ending position, and family name and superfamily name of MITE, which are delimitated by vertical bars. There are only 17 MITE sequences shown in the figure as the other two accessions do not have the homologous MITE in the proximity region, potentially due to the differential transposition activity between different Arabidopsis accessions. Specifically, the MITE sequence in the first row (from the *zu* accession) is the only canonical MITE possessing perfect TSD and TIR pairs, while the other 16 MITE sequences do not.

We then used RepeatMasker, a homology-based approach, to detect all non-canonical MITEs (*i.e*., potential diverse and partial MITEs) in 19 accession genomes. Consequently, we have identified a total of 343485 MITEs in all 19 accessions, among which 19.31% (*i.e*., 66339) belonged to previously known or classified MITE families, whereas 80.69% (*i.e*., 277146) belonged to novel or unclassified MITEs (Table 1). Within the classified MITEs, 39 families and 4 superfamilies were detected, as the family information was inherited from the above 212 annotated representative sequences. The basic sequence features were studied and illustrated in Supplementary Table 1. As described, different superfamilies possessed different MITE lengths and GC contents on average. Interestingly, the overall GC content of *Tc1/Mariner* seems to be even lower than other MITE superfamilies. Regarding the sequence integrity, most MITEs did not have perfect or near perfect TIR and TSD sequences among different accessions, and sometimes no obvious TIR and TSD fragments were even found. For example, a MITE in the *zu* accession had a perfect TIR and TSD sequences in chromosome 1 from the position 2323922 to 2324186 where no other genic unit was annotated nearby. Also, the homology search suggested this MITE was only detected in 17 accessions in the same region, including the canonical MITE in *zu* accession and the non-canonical MITEs with imperfect TSD/TIR structure in the other 16 accessions. MITEs were not detected in *bur* and *wu*, indicating that transposition had occurred in these two accessions. As expected, these 16 detected MITEs belong to the same family as the one in the *zu* accession. Both ends of the 17 MITE sequences were aligned in MEGA6^57^ with ClustalW and the sequence alignment was displayed in Fig. 2. Additionally, to validate the authenticity of the MITEs detected in our study, the *A. thaliana* MITEs in P-MITE (the accession used in the P-MITE database is *col*) was compared with the *col* MITEs in our dataset. Among all of the 3245 MITE sequences detected in Arabidopsis *col* accession in P-MITE, 94.3% of them were verified by our *col* dataset. While the actual false positive rate for this discovery analysis of novel MITE sequences is unknown, we have attempted to minimize the number of false positives by using detectMITE since differently from other similar tool, detectMITE incorporates multiple methods to minimize false positives^43^.

**Table 1:** Summary of MITEs in all 19 accessions.

Accession	Previously Classified MITEs									Unclassified
	Chromosome					Superfamily				
	Chr1	Chr2	Chr3	Chr4	Chr5	Mutator	PIF/Harbinger	Tc1/Mariner	hAT	
*bur*	851	729	584	606	692	628	700	975	1159	14581
*can*	852	738	610	588	672	634	722	943	1161	14292
*col*	915	766	635	655	749	691	755	1057	1217	15402
*ct*	867	734	593	587	687	636	713	965	1154	14561
*edi*	840	738	610	603	695	652	708	944	1182	14523
*hi*	854	743	596	604	693	644	703	993	1150	14803
*kn*	851	722	596	600	684	624	688	981	1160	14424
*ler*	846	728	619	596	670	641	693	977	1148	14365
*mt*	845	736	597	598	683	627	706	973	1153	14549
*no*	849	727	603	594	692	632	704	974	1155	14711
*oy*	847	741	592	608	682	639	704	984	1143	14389
*po*	890	737	601	615	695	642	730	999	1167	14746
*rsch*	860	755	610	608	699	620	732	983	1197	14409
*sf*	852	727	607	594	678	616	705	972	1165	14631
*tsu*	854	730	612	589	692	624	689	981	1183	14651
*wil*	871	730	589	597	683	641	697	982	1150	14304
*ws*	852	723	619	592	697	628	707	974	1174	14608
*wu*	864	753	604	587	683	630	707	988	1166	14609
*zu*	863	733	613	598	691	642	691	965	1200	14588

### Distribution of MITE families

**Figure 3:**
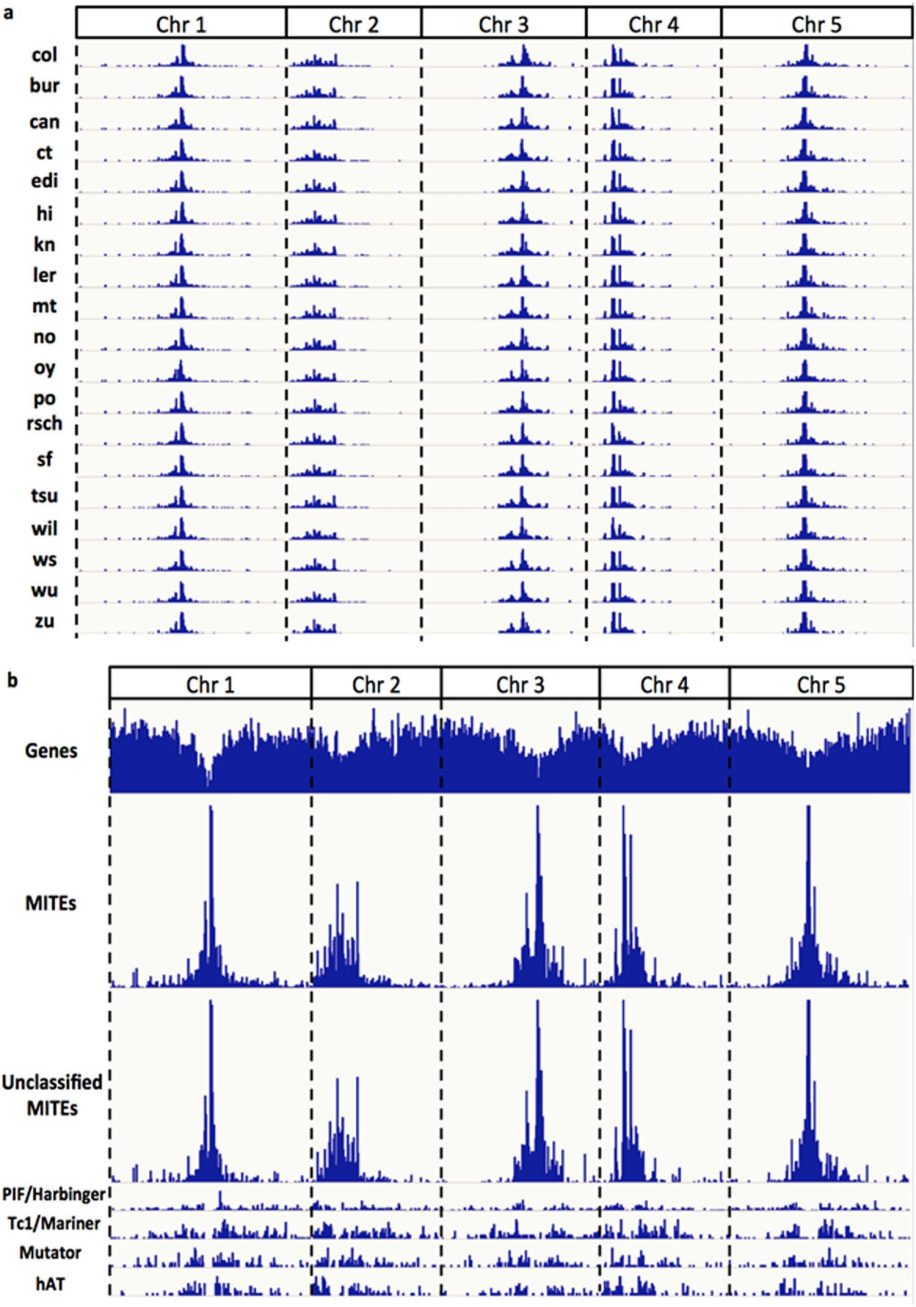
MITE distributions along the five chromosomes in *Arabidopsis thaliana*. (**a**) Relative locations and abundances of MITEs in all 19 accessions. (**b**) The comparison of the distributions of different MITE superfamilies in *col*. The top track shows the gene density along 5 chromosomes (Chr1 to Chr5), where troughs indicate heterochromatic centromere regions. The second top track represents the distribution of the MITE population along chromosomes. Depending on whether a specific MITE sequence was defined previously or not, MITEs are categorized into unclassified and classified MITEs, and classified MITEs are further grouped into four super-families (i.e., *PIF/Harbinger*, *Tc1/Mariner*, *Mutator* and *hAT*) based on their sequence characterizations. The distributions of MITEs in the four superfamilies are displayed in the rest of the tracks respectively.

Studying the distribution of MITEs may reveal the preference of MITE transposition, and possibly explain the underlying transposition mechanism. The MITE distributions in 19 accessions were summarized in Table 1. MITE densities in the five chromosomes showed a similar proportion across different accessions. MITEs also had a very similar distribution pattern among different accessions (Fig. 3a). They formed major peaks of frequency at the heterochromatin regions, where no or few active genes were found, while the frequency gradually decreased towards both ends of a chromosome. Further investigation of the MITEs by comparing with previously characterized MITE superfamilies revealed that the overall distribution of MITEs was greatly determined by the unclassified MITEs, which accounted for more than 80% of the total MITEs. Although the majority of unclassified MITEs were concentrated in the heterochromatin regions, numerous MITEs, especially those previously classified MITEs, were found located within euchromatin regions. In contrast to the unclassified group, the classified MITEs were fairly depleted in the heterochromatin regions, but enriched in the peripheral heterochromatin regions. As the patterns in all accessions were similar, the *col* accession was selected as the representative one in Fig. 3b.

### The interactions between genes and MITEs

The expected interactions between genes and MITEs are abundant, considering 1) MITEs are in close proximity to genes compared to non-MITE transposable elements^2^, and 2) *A. thaliana* is a highly compacted genome. To examine the direct influence of MITEs on gene structure, the insertion sites of MITEs were inspected and compared in all genomes of the 19 accessions. All MITE-gene interactions were classified into two groups, that the MITE is either solely embedded in an individual sub-region of a gene or spanned over multiple sub-regions within a gene. Focusing first on the MITE-gene interactions between MITEs and known genes, we found 3065 MITEs were embedded in a single sub-region and 2647 spanned sub-regions. Since MITEs are largely considered as foreign elements by host genomes, among the MITE-gene interactions in the former group, in which the MITE is solely embedded in an individual sub-region, 99.7% (3057) of them were found inside individual introns. While interestingly, among the latter MITE-gene interaction group where MITEs spanned over multiple sub-regions in gene, 87.2% (2308) of them were located in the last exon (*i.e*., consisting of partial CDS and partial 3′ UTR in genes). In other words, these MITE sequences may directly contribute to the stop codon of genes. In summary, the former cases suggest that the MITEs were recognized and spliced out using introns to avoid mutation, while the latter case suggests that the MITEs were strongly influential in modifying the structure of 3′ UTRs in genes, potentially introducing new ends for genes by providing poly(A) signals for new polyadenylation sites. A following analysis to compare the characterizations of the two MITE-gene interaction groups suggested no significant difference between the features of MITEs. Among the 3057 intron-contained MITEs, 405 are from the *hAT*, 390 from the *Mutator*, 520 from the *PIF/Harbinger*, 666 from the *Tc1/Mariner* and 1076 from the unclassified superfamily. While among the 2308 MITEs located in the 3′ UTR region, 303 are from the *hAT*, 266 from the *Mutator*, 445 from the *PIF/Harbinger*, 793 from the *Tc1/Mariner* and 501 from the unclassified superfamily. Moreover, the spanning patterns of MITEs were mostly consistent in different accessions, such that if a MITE was found in the specific region of the host gene, then all other accessions with the MITE followed the same pattern. Additionally, there were a total of 29700 MITE-gene interactions between unclassified MITEs and genes, but the majority of them (27694) were linked with novel genes, indicating a strong association between unclassified MITEs and novel genes.

### Transpositions of MITE has little influence on gene expression

**Figure 4:**
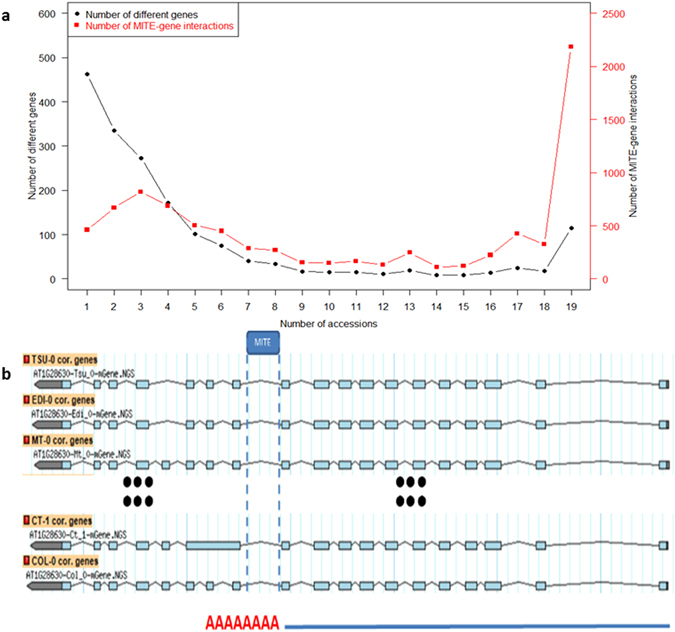
Summary of the MITE-gene interactions. (**a**) The shared MITE-gene interaction in 19 Arabidopsis accessions. The X-axis represents the number of different accessions that share the same MITE-gene interaction. The left Y-axis (black) represents the number of MITE-related genes. The MITE-related genes are defined as those that contain or are adjacent to the same MITEs in at least one accession. The right Y-axis (red) presents MITE-gene interaction values, which are defined and quantified as the production of multiplying the number of MITE-related genes with the number of accessions having the same MITE-related genes. For instance, the first MITE-gene interaction value is 462 (weak interaction), which means there are 462 MITE-related genes present only in one accession. The last MITE-gene interaction value is 2185 (strong interaction), which means there are 115 MITE-related genes common in all 19 accessions. (**b**) The snapshot of MITE-gene interactions associated with gene AT1G28630.

The frequent transposition activity of MITEs and the variation of genomes in different accessions make the comparative analyses of homologous MITEs in our study difficult to implement. To work around the problem and narrow down the possible homologous MITEs, we used genes as the markers of genomic locations. Specifically, we first identified the homologous genes that contained or were adjacent to the same MITEs (i.e., ≤ ±40 *nt* between ends of genes and MITEs), which we called MITE-related genes. Utilizing the MITE-gene interactions between classified MITEs and known genes as a measurement, we summarized and quantified these MITE transposition activities across accessions in Fig. 4a. For instance, 462 MITE-related genes had a weak MITE-gene interaction, because such interaction was only shown in a single accession (i.e., 462 genes × 1 accession = 462). In contrast, 115 MITE-related genes showed strong MITE-gene interactions because such interaction appeared in all 19 accessions (115 genes × 19 accessions = 2185). As shown, the number of MITE-related genes varied among the 19 accessions. The data points on the left side suggested weak MITE-gene interactions, which meant the MITE-related genes appeared only in one or a few accessions, likely to be a newly formed MITE-gene interaction. The data points on the right suggested stronger MITE-gene interactions, which indicated the MITE-related genes were shared in most accessions, implying these MITEs may be functionally conserved among different accessions. Possible enriched functions for the genes with strong and weak MITE-gene interactions were studied separately using DAVID web service^58^, but the result suggested no significant function in any of the two groups. Collectively, the transposition activities of MITEs seem not to have come from interactions with specific genomic and environmental factors, but are more likely to be from a random process during evolution.

Genes with MITEs in proximity have been reported with an overall lower expression^6, 59^, possibly caused by the silencing impact from MITEs. Thus MITEs are normally regarded to down regulate the gene expression. To note, these previous studies were conducted by comparing gene expressions between genes with and without MITEs in proximity using genome-wide transcriptomic data in single species. However, no research has been devoted to understand the direct impact of the transposition activities of MITEs on individual genes across different accessions of the same species regarding gene expression. Integrated with processed transcriptomic data from the original study^48^, our work actually provided a perfect set of data to examine the expressions of the homologous gene with or without a MITE among accessions to answer the question in a intraspecies fashion. The expression levels of genes from all 19 accessions were first collected^48^ if a known MITE-gene interaction occurred in any of the accessions for the specific gene. For each gene, accessions were categorized into two groups based on the presence of a MITE-gene interaction within the host gene (*i.e*., the group of genes with the MITE-gene interaction and the group of genes without the MITE-gene interaction). The genes with at least one MITE interaction were considered and the average RPKM value was used to represent the relative gene expression. For the overall gene expression in all selected genes and in all 19 accessions, the comparison between the accessions with and without a MITE in proximity indicated significant difference (P-value = 1.09e-05), as the average expression value for genes with a MITE in proximity was 16.7, while the genes without a MITE was 11. Unfortunately, at individual gene level, the expression levels of the individual genes were also compared individually but no gene was detected with significant difference in expression level between the two accession groups (accessions with and without MITE-gene interactions for the individual gene).

## Discussion

MITEs are known as essential components in eukaryotic genomes causing direct influence on genome size and genome structure. Over the past decade, studies have been conducted to identify, characterize and compare the MITE sequences among organisms to understand the impact of MITEs on genome evolution. However, systematic and comprehensive studies are still lacking. Providing insights for the evolutionary process and the consequent influence of MITEs upon species diversity and phenotype plasticity in *A. thaliana*, we conducted a new approach of identifying MITEs in many different accessions within the same species (Fig. 1), which greatly increased the total population of putative MITEs. Further, we created a database of MITEs in *A. thaliana* by cataloging and determining their genetic configurations, and also explored the interconnections of MITEs with gene structure and expression of host genes. The 19 *A. thaliana* accessions studied in our project were parents of the MAGIC lines, which are intentionally selected to maximize phenotypic variation and geographical diversity, as lines are appropriate representatives of both phenotypic and genotypic variation in the global *A. thaliana* population^49, 60^.

Different from conventional string search-and-comparison methods with quadratic runtime complexity, in our study, detectMITE implemented the algorithm using numeric calculation and manipulation with linear time complexity that also enables more accurate and exhaustive search in MITE detection comparing to other tools^43^. Another significant improvement of our pipeline of MITE detection was that we pooled and clustered all putative MITEs from the 19 accessions together. As an example shown in Fig. 2, only the MITE sequence in the *zu* accession displayed perfect TIR and TSD sequence fragments, while this was not the case for the other accessions. Theoretically, these corresponding MITEs in the other accessions will be dismissed by the de novo detection, and the entire lineage of this MITE sequence could be missed if genomes were analyzed separately. Clearly, our approach of pooling all *de novo* detected MITEs for individual accessions for the homologous search of canonical MITEs greatly improved the performance of MITE detection, which could be easily applied to other species in the future. Also, although a MITE is featured by flanking TSDs, TSDs are not a part of the MITE sequence, as the transposition of a MITE in the genome normally does not include TSDs. Later, CD-HIT was applied to cluster all detected canonical MITE sequences into 212 canonical MITEs groups. The superfamily and family information were inherited from the P-MITE database accordingly. To note, the representatives of 6 MITE clusters (out of 212) were annotated with a family name under a plant species other than Arabidopsis, but closely related species, such as *Brassica rapa* and *Manihot esculenta*. The association may indicate a strong conservation of the MITEs in different plant species where most MITE amplifications are likely formed after specification, considering there were a total of 9421 families and 43 plant species in the P-MITE database. Afterwards, a homologous search based on the pooled and clustered canonical MITE sequences was conducted to find the entire MITE population in the genomes.

Consequently, a total of 343485 MITE sequences, including perfect, diverse and partial ones, were identified in the 19 accessions. Slightly different from a previous study that MITEs in the five chromosomes showed a similar density with respect to the chromosome sizes^13^, the MITEs in chromosome 2 and chromosome 4 in our study presented a overall higher density, and the reason is unclear.

Compared to the *A. thaliana* records, *i.e*., 3245 MITEs detected for *col*, in the P-MITE database, this was a huge increase in our study, averaging 18078 MITEs in individual accession. Among the MITEs in the P-MITE database, 94.3% were covered by our data, which ensures the authenticity of our putative MITE sequences. Surprisingly, more than 80% of MITEs were classified into the unclassified group, as no previously recorded homologous sequence was found from the P-MITE database. A further analysis of these unclassified MITE sequence indicated that many of them were short sequences, typically less than 100 *nt*. Also these unclassified MITEs were spatially enriched in the heterochromatin regions, which may imply that most unclassified MITEs were inactive in the genome. Further comparison on the level of epigenetics marks on unclassified MITEs located in both heterochromatin and euchromatin regions may provide more insights for their transposition activities. Lastly, a strong association was found between the unclassified MITEs with novel genes (*ab initio* predicted gene based on the transcriptomics data in the original study, which were not recorded in the TAIR10) in all accessions. The authenticities of these novel genes are still arguable, but the phenomenon at least indicates that these MITEs were highly transcribed, and could be detected by RNA-Seq analysis.

MITEs are frequently inserted in the proximity of genes, thus having a high chance of altering their structure and expression. MITE sequences may provide transcription start sites, poly(A) signals, exons, and splice junctions for plant genes^29^. It has been reported that more than 7000 MITEs in rice cultivar Nipponbare were transcribed, and approximately half of them were present in transcripts with coding sequences^9^. MITE sequences were also frequently found in the EST database of other plant species^31^. The co-transcription of MITEs and adjacent genes may indicate an important role MITEs play in regulating gene expression. Meanwhile, the well-annotated genome of all 19*A. thaliana* accessions provided a unique resource for studying the transposition activities of MITEs along the evolutionary divergence of these accessions. As described in Fig. 4a, in total 8390 interactions were found between classified MITEs and known genes in all 19 accessions. The genes with strong MITE-gene interaction indicates the transposition activities of their associated MITEs were probably established before the specification *A. thaliana* was formed and that the MITEs remained in genes at the insertion loci with potential functional importance. On the other hand, the genes with weak MITE-gene interaction implied that their associated MITEs were newly transferred into the host genes after divergence of ecological accessions through evolution. Of course, other hypotheses might also be possible, which are worthy of further exploration.

More interestingly, in MITE-related genes, MITEs tend to exist in either introns or 3′ UTR exons, which might possess important regulatory roles in gene expression. As shown in Fig. 4b, a MITE that is common among 16 of the 19 accessions, with a high MITE-gene interaction value (*i.e*., a strong MITE-gene interaction), was found inside the intron. There were 5712 classified MITEs (not unclassified or novel ones as shown in Fig. 3) that were contained within or adjacent to genes annotated in TAIR10 across the 19 accessions. Among them, 53.52% (3057) of MITEs were found inside an intron of genes, while 40.41% (2308) of the MITEs spanned over multiple locations in the last exon (i.e., consisting of a partial CDS and a partial 3′ UTR in genes). Interestingly, an alternative polyadenylation site, located in the upstream region of the MITE (see Fig. 4b), was also detected and supported by RNA-Seq data. It turns out that 99.7% (3057/3065) of MITEs located in a single sub-genic region were located in introns. Additionally, 87.2% (2308/2647) of MITEs whose location spanned over multiple sub-genic regions also spanned the last exon to the 3′ UTR. The former case suggests that the foreign element (MITE) was recognized and spliced out using an intron to avoid mutation by the host genome, while the latter case suggests that MITEs were strongly influential in modifying the three prime ends of genes, potentially introducing a great number of new gene ends by providing poly(A) signal for polyadenylation. MITE sequences possess relatively low GC content, which actually provides an appropriate poly(A) signal for the polyadenylation machinery to target.

There are two potential MITE-regulated polyadenylation cases: 1) the transposition of a MITE introduces a strong poly(A) signal that directly shortens the gene length, and 2) the transposition of a MITE introduces a relatively weak poly(A) signal to the gene compared to the gene’s original poly(A) signal, and therefore results in an alternative poly(A) site, which normally is a non3UTR poly(A) site^61^, as exemplified by Fig. 4b. Additionally, a previous study of MITE family *mPifvine-3.1* in grapevine shows that they were concentrated in 3′ UTR regions, which is in contrast with other *mPifvine* MITEs elements^62^. The preferential localization of *mPIfvine-3.1* suggests an underlying hidden mechanism of the transposition for these specific MITE elements.

MITEswere normally regarded to impede gene expression, since the genes with MITE-gene interaction were reported to have an overall lower expression than the ones without^6, 59^, which is explained by the potential silencing impact a MITE brought into its host gene. On the other hand, the impact of a MITE in individual gene regulation has been occasionally reported. For instance, the MITE insertion into promoter region of *Ubiqutin2* gene increased the gene expression level in rice, and such enhancement effect of expression could be also neutralized by DNA methylation upon the MITE sequence^36^. Another study suggests that a MITE operates as a *cis*-acting element to up-regulate *SbMATE*, which was located upstream of the *sbMATE* gene, introducing a positive correlation between the presence of the repeat structure and aluminum tolerance^37^. A MITE insertion in the 3′ UTR of a small heat shock protein gene (*TaHSP16.9–3A*) in wheat was shown to increase the transcription level of its host gene under heat stress, compared to the other wheat haplotypes without the MITE insertion, resulting in heat tolerance^38^. However, the explanation of increased expression of the host gene is still controversial. Different mechanisms have been proposed under individual gene circumstances, depending on the relative location of the MITE or even the characterization of the MITE family. Additionally, Quadrana and his colleagues recently reported in a TEs study involved hundreds of *Arabidopsis thaliana* accessions, that TEs have an equal opportunity of enhancing or repressing gene expression, but the underlying mechanism is still unclear^63^. In our study, we found that the overall gene expression of genes with a MITE-gene interaction hold a higher expression, compared to the ones without a MITE in proximity, although no individual gene was detected with significantly different expression between the accession groups with and without MITEs. In summary, future explorations of the impact of MITEs on individual genes and the possible mechanism are needed to obtain a deeper understanding of the regulatory function of MITEs.

## Electronic supplementary material

Supplementary Table 1

## References

[1] Bureau TE, Wessler SR (1992). Tourist: a large family of small inverted repeat elements frequently associated with maize genes. Plant Cell.

[2] Fattash I (2013). Miniature inverted-repeat transposable elements: discovery, distribution, and activity. Genome/Natl. Res. Counc. Canada.

[3] Lander ES (2001). Initial sequencing and analysis of the human genome. Nature.

[4] Mouse Genome Sequencing Consortium *et al*. Initial sequencing and comparative analysis of the mouse genome. *Nature* **420**, 520–62 (2002). 10.1038/nature01262 12466850

[5] Meyers BC, Tingey SV, Morgante M (2001). Abundance, distribution, and transcriptional activity of repetitive elements in the maize genome. Genome Res.

[6] Feng Y (2003). Plant MITEs: useful tools for plant genetics and genomics. Genomics. Proteomics Bioinformatics.

[7] Paterson AH (2009). The Sorghum bicolor genome and the diversification of grasses. Nature.

[8] Nouroz F, Noreen S, Heslop-Harrison JS (2015). Evolutionary genomics of miniature inverted-repeat transposable elements (MITEs) in Brassica. Mol. Genet. Genomics.

[9] Lu C (2012). Miniature inverted-repeat transposable elements (MITEs) have been accumulated through amplification bursts and play important roles in gene expression and species diversity in Oryza sativa. Mol. Biol. Evol..

[10] Casa AM (2000). The MITE family heartbreaker (Hbr): molecular markers in maize. Proc. Natl. Acad. Sci. USA.

[11] Casacuberta E, Casacuberta JM, Puigdomènech P, Monfort A (1998). Presence of miniature inverted-repeat transposable elements (MITEs) in the genome of Arabidopsis thaliana: characterisation of the Emigrant family of elements. Plant J..

[12] Charrier B (1999). Bigfoot. a new family of MITE elements characterized from the Medicago genus. Plant J..

[13] Chen J, Hu Q, Zhang Y, Lu C, Kuang H (2014). P-MITE: a database for plant miniature inverted-repeat transposable elements. Nucleic Acids Res.

[14] Wicker T (2007). A unified classification system for eukaryotic transposable elements. Nat. Rev. Genet..

[15] Kapitonov VV, Jurka J (2008). A universal classification of eukaryotic transposable elements implemented in Repbase. Nat. Rev. Genet..

[16] Dai S (2015). Widespread and evolutionary analysis of a MITE family Monkey King in Brassicaceae. BMC Plant Biol.

[17] Sampath P, Yang T-J (2014). Miniature Inverted-repeat Transposable Elements (MITEs) as Valuable Genomic Resources for the Evolution and Breeding of Brassica Crops. Plant Breed. Biotechnol.

[18] Sampath P (2014). Genome-Wide Comparative Analysis of 20 Miniature Inverted-Repeat Transposable Element Families in Brassica rapa and B. oleracea. PLoS One.

[19] He Q, Ma Z, Dang X, Xu J, Zhou Z (2015). Identification, Diversity and Evolution of MITEs in the Genomes of Microsporidian Nosema Parasites. PLoS One.

[20] Feschotte C, Mouchès C (2000). Evidence that a family of miniature inverted-repeat transposable elements (MITEs) from the Arabidopsis thaliana genome has arisen from a pogo-like DNA transposon. Mol. Biol. Evol..

[21] Zhang X (2001). P instability factor: an active maize transposon system associated with the amplification of Tourist-like MITEs and a new superfamily of transposases. Proc. Natl. Acad. Sci. USA.

[22] Feschotte C, Osterlund MT, Peeler R, Wessler SR (2005). DNA-binding specificity of rice mariner-like transposases and interactions with Stowaway MITEs. Nucleic Acids Res..

[23] Loot C, Santiago N, Sanz A, Casacuberta JM (2006). The proteins encoded by the pogo-like Lemi1 element bind the TIRs and subterminal repeated motifs of the Arabidopsis Emigrant MITE: consequences for the transposition mechanism of MITEs. Nucleic Acids Res..

[24] Hancock CN, Zhang F, Wessler SR (2010). Transposition of the Tourist-MITE mPing in yeast: an assay that retains key features of catalysis by the class 2 PIF/Harbinger superfamily. Mob. DNA.

[25] Hancock CN (2011). The rice miniature inverted repeat transposable element mPing is an effective insertional mutagen in soybean. Plant Physiol..

[26] Yang G, Zhang F, Hancock CN, Wessler SR (2007). Transposition of the rice miniature inverted repeat transposable element mPing in Arabidopsis thaliana. Proc. Natl. Acad. Sci.

[27] Makarevitch I (2015). Transposable Elements Contribute to Activation of Maize Genes in Response to Abiotic Stress. PLoS Genet..

[28] Yang G, Nagel DH, Feschotte C, Hancock CN, Wessler SR (2009). Tuned for transposition: molecular determinants underlying the hyperactivity of a Stowaway MITE. Science.

[29] Oki N (2008). A genome-wide view of miniature inverted-repeat transposable elements (MITEs) in rice, Oryza sativa ssp. japonica. Genes Genet. Syst..

[30] Tu Z (1997). Three novel families of miniature inverted-repeat transposable elements are associated with genes of the yellow fever mosquito, Aedes aegypti. Proc. Natl. Acad. Sci. USA.

[31] Kuang H (2009). Identification of miniature inverted-repeat transposable elements (MITEs) and biogenesis of their siRNAs in the Solanaceae: new functional implications for MITEs. Genome Res..

[32] Sarilar V, Marmagne A, Brabant P, Joets J, Alix K (2011). BraSto, a Stowaway MITE from Brassica: recently active copies preferentially accumulate in the gene space. Plant Mol. Biol..

[33] Santiago N, Herráiz C, Goñi JR, Messeguer X, Casacuberta JM (2002). Genome-wide analysis of the Emigrant family of MITEs of Arabidopsis thaliana. Mol. Biol. Evol..

[34] Lu C (2012). Miniature Inverted-Repeat Transposable Elements (MITEs) Have Been Accumulated through Amplification Bursts and Play Important Roles in Gene Expression and Species Diversity in Oryza sativa. Mol. Biol. Evol..

[35] Ha M, Kim VN (2014). Regulation of microRNA biogenesis. Nat. Rev. Mol. Cell Biol..

[36] Yang G (2005). A two-edged role for the transposable element Kiddo in the rice ubiquitin2 promoter. Plant Cell.

[37] Magalhaes JV (2007). A gene in the multidrug and toxic compound extrusion (MATE) family confers aluminum tolerance in sorghum. Nat. Genet..

[38] Li J, Wang Z, Peng H, Liu Z (2014). A MITE insertion into the 3′-UTR regulates the transcription of TaHSP16.9 in common wheat. Crop J.

[39] Bender J, Fink GR (1995). Epigenetic control of an endogenous gene family is revealed by a novel blue fluorescent mutant of Arabidopsis. Cell.

[40] Luff B, Pawlowski L, Bender J (1999). An inverted repeat triggers cytosine methylation of identical sequences in Arabidopsis. Mol. Cell.

[41] Murukarthick J (2014). BrassicaTED - a public database for utilization of miniature transposable elements in Brassica species. BMC Res. Notes.

[42] Yang G (2013). MITE Digger, an efficient and accurate algorithm for genome wide discovery of miniature inverted repeat transposable elements. BMC Bioinformatics.

[43] Ye C, Ji G, Liang C (2016). detectMITE: A novel approach to detect miniature inverted repeat transposable elements in genomes. Sci. Rep.

[44] Han Y, Wessler SR (2010). MITE-Hunter: a program for discovering miniature inverted-repeat transposable elements from genomic sequences. Nucleic Acids Res..

[45] Chen J, Lu C, Zhang Y, Kuang H (2012). Miniature inverted-repeat transposable elements (MITEs) in rice were originated and amplified predominantly after the divergence of Oryza and Brachypodium and contributed considerable diversity to the species. Mob. Genet. Elements.

[46] Dubreuil-Tranchant C (2011). Site-Specific Insertion Polymorphism of the MITE Alex-1 in the Genus Coffea Suggests Interspecific Gene Flow. Int. J. Evol. Biol..

[47] Wright SI, Agrawal N, Bureau TE (2003). Effects of recombination rate and gene density on transposable element distributions in Arabidopsis thaliana. Genome Res..

[48] Gan X (2011). Multiple reference genomes and transcriptomes for Arabidopsis thaliana. Nature.

[49] Kover PX (2009). A Multiparent Advanced Generation Inter-Cross to fine-map quantitative traits in Arabidopsis thaliana. PLoS Genet..

[50] Lamesch P (2012). The Arabidopsis Information Resource (TAIR): improved gene annotation and new tools. Nucleic Acids Res.

[51] Wu TD, Watanabe CK (2005). GMAP: a genomic mapping and alignment program for mRNA and EST sequences. Bioinformatics.

[52] Fu L, Niu B, Zhu Z, Wu S, Li W (2012). CD-HIT: accelerated for clustering the next-generation sequencing data. Bioinformatics.

[53] Chen, N. Using RepeatMasker to identify repetitive elements in genomic sequences. *Curr. Protoc. Bioinformatics* **4**, 4.10 (2004). 10.1002/0471250953.bi0410s05 18428725

[54] Raney BJ (2014). Track data hubs enable visualization of user-defined genome-wide annotations on the UCSC Genome Browser. Bioinformatics.

[55] Robinson JT (2011). Integrative genomics viewer. Nat. Biotechnol..

[56] Mann HB, Whitney DR (1947). On a Test of Whether one of Two Random Variables is Stochastically Larger than the Other. Ann. Math. Stat..

[57] Tamura K, Stecher G, Peterson D, Filipski A, Kumar S (2013). MEGA6: Molecular Evolutionary Genetics Analysis version 6.0. Mol. Biol. Evol..

[58] Huang DW, Lempicki RA, Sherman BT (2009). Systematic and integrative analysis of large gene lists using DAVID bioinformatics resources. Nat. Protoc..

[59] Slotkin RK, Martienssen R (2007). Transposable elements and the epigenetic regulation of the genome. Nat. Rev. Genet..

[60] Weigel D, Mott R (2009). The 1001 Genomes Project for Arabidopsis thaliana. Genome Biol..

[61] Guo C, Spinelli M, Liu M, Li QQ, Liang C (2016). A Genome-wide Study of ‘Non-3UTR’ Polyadenylation Sites in Arabidopsis thaliana. Sci. Rep..

[62] Benjak A, Boue S, Forneck A, Casacuberta JM (2010). Recent amplification and impact of MITEs on the genome of grapevine (Vitis vinifera L.). Genome Biol. Evol..

[63] Quadrana, L. *et al*. The Arabidopsis thaliana mobilome and its impact at the species level. *Elife* **5** (2016). 10.7554/eLife.15716 PMC4917339 27258693

